# Association of Serum Biomarkers with Outcomes and Treatment Success of Inhaled Hyaluronan in COVID19

**DOI:** 10.1007/s00408-025-00858-8

**Published:** 2025-11-27

**Authors:** Jane S. Der, Claudio Pedone, Flavia Galdi, Christopher A. McGee, Raffaele Antonelli Incalzi, Stavros Garantziotis

**Affiliations:** 1 DLH Corp., LLC, Bethesda, MD USA; 2 Operative Research Unit of Geriatrics, Fondazione Policlinico Campus Bio-Medico, and Research Unit of Geriatrics, Department of Medicine and Surgery, Università Campus Bio-Medico di Roma, Rome, Italy; 3 Operative Research Unit of Internal Medicine, Fondazione Policlinico Campus Bio-Medico, Rome, IT Italy; 4 Division of Intramural Research, National Institute of Environmental Health Sciences, Research Triangle Park, NC USA; 5 Research Unit of Geriatrics, Department of Medicine and Surgery, Università Campus Bio-Medico di Roma, Rome, Italy

**Keywords:** Hyaluronan, Viral pneumonia, CXCL10, Outcomes

## Abstract

Viral pneumonia causes significant morbidity and mortality worldwide. However, there is limited ability to predict outcomes and treatment responses. We analyzed data from a recently published placebo-controlled trial of inhaled high molecular weight hyaluronan (HMWHA) in 146 patients with severe COVID-19 pneumonia, to determine whether admission cytokines and demographic information is associated with disease outcomes and response to HMWHA treatment. We found that serum levels of CXCL10 are strongly associated with both endpoints. Our data thus identify CXCL10 as a possible predictor of viral pneumonia outcome and response to anti-inflammatory treatment.

## Introduction

Viral pneumonia is a major cause of adult respiratory distress syndrome (ARDS), morbidity and mortality worldwide [1]. Treatments for viral pneumonia are still lacking, and our ability to predict clinical outcomes is limited. There is thus a critical need to establish biomarkers that stratify patients depending on adverse outcome risk, and can predict treatment responses.

High molecular weight hyaluronan (HMWHA), a glycosaminoglycan sugar that is naturally found in connective tissue and is generally defined as being > 500 kDa [2], has powerful anti-inflammatory properties [2, 3] which make it a possible candidate as a novel biologic in lung disease [4, 5]. We recently reported that HMWHA ameliorates COPD exacerbations [6] and viral pneumonia, including COVID19 [7]. However, not all COVID19 patients benefitted from HMWHA treatment. We reanalyzed the results from our clinical trial addressing two questions: are blood cytokines on admission associated with: (a) COVID19 outcomes; and (b) response to HMWHA treatment?

## Methods

Details on the clinical trial (NCT04830020) were published previously [7]. Briefly, patients admitted for severe COVID19, needing supplemental O_2_ but no ventilator support, received state-of-the-art treatment and in addition either HMWHA (Yabro^®^, IBSA Pharmaceuticals, Switzerland) or placebo, twice daily by nebulizer, for 10 days or until discharge, whichever came first. Blood was drawn on admission and stored at -80 °C until analyzed. Based on previously published literature on cytokines associated with viral pneumonia [8], we analyzed blood IL6, CXCL8, CXCL10, CCL2, CCL3, IL10, and TNFα (Bio-plex Pro multiplex panel, Bio-Rad, Hercules, CA). Our cohort included 146 patients (placebo, *N* = 75; HMWHA, *N* = 71) [7]. The analyzed outcomes were (a) time to hospital discharge (henceforth called “discharge”); (b) time to liberation from supplemental O_2_ (“O_2_”); and (c) composite outcome consisting of both discharge or O_2_, whichever came first (“recovery”, as defined previously [9]). We evaluated cytokine profiles for COVID19 outcome and treatment effect associations using uni- and multivariate Cox proportional hazard models, with age, gender, PaO_2_/FiO_2_, and biomarkers as our variables. Given the relatively low number of study patients, we took an exploratory approach to our analyses. Therefore, p-values < 0.1 were considered suggestive of statistical significance for this analysis. This threshold was chosen to reduce the risk of Type II error in detecting potential associations that warrant further evaluation in larger, confirmatory trials.

## Results

We performed principal component analysis of measured cytokine concentrations to simplify the data into a smaller set while still capturing significant patterns. Principal components were chosen based on eigenvectors of the covariance matrix, and their importance determined by eigenvalues, which represent the variance that is explained by each component. The first principal component (PC1) explained 38% of the variance, while PC1 and PC2 together explained 54% of the variance in the placebo patient dataset. PC1 was a weighted average of all cytokines (i.e., all coefficients were positive with varying magnitude, with CCL2 and TNFα being weighted the heaviest). PC2 was dominated by CCL3, IL8 (positive coefficients), CXCL10 and IL6 (negative coefficients). We computed two scores for each patient:

$$\begin{aligned} & {\text{PC}}1\;{\text{Score}} = 0.{\text{449558}}*{\text{lnIL6}} + 0.{\text{329379}}*{\text{lnIL8}} \\ & \quad + 0.0{\text{814}}0{\text{9}}*{\text{lnIL1}}0 + 0.{\text{333367}}*{\text{ lnCXCL1}}0 \\ & \quad + 0.{\text{532847}}*{\text{ lnCCL2}}\, + \,0.{\text{291714}}*{\text{lnCCL3}} \\ & \quad + 0.{\text{45}}0{\text{136}}*{\text{lnTNF}}\alpha . \\ \end{aligned} $$

$$\begin{aligned} & {\text{PC}}2\;{\text{Score}} = - 0.{\text{117281}}*{\text{lnIL6}} + 0.{\text{271847}}*{\text{lnIL8}} \\ & \quad + 0.0{\text{91415}}*{\text{lnIL1}}0{\text{ }} - 0.{\text{615}}0{\text{19}}*{\text{lnCXCL1}}0 \\ & \quad - 0.0{\text{9}}0{\text{5}}0{\text{2}}*{\text{lnCCL2}} + 0.{\text{7194}}0{\text{6}}*{\text{lnCCL3}} \\ & \quad {-}0.00{\text{193}}0*{\text{lnTNF}}\alpha . \\ \end{aligned} $$

For each patient, cytokine concentrations and PC scores were categorized as being above- or below-median, based on cut-off points computed from the placebo patients. In multivariate Cox proportional hazard models for the association of cytokine profiles with COVID19 outcome (question 1), we found that older age (> 62 years, cut-off chosen for increased power based on the age distribution of this cohort) and more severe hypoxia (PaO_2_/FiO_2_ < 200) were significantly associated with all outcomes. CXCL10 and PC2 were significantly associated with recovery and O_2_ (Table 1). In univariate models, CXCL10 and PC2 were significantly associated with recovery, and CXCL10 was significantly associated with O_2_ (Table 2). CXCL10 values above median and PC2 scores below median signified worse outcomes. Besides CXCL10, no other cytokines predicted outcomes when analyzed alone.

We then evaluated associations of CXCL10, PC1 and PC2 with the response to HMWHA treatment (question 2), using univariate Cox proportional hazard models for HMWHA vs. placebo in the above- and below-median subsets for these biomarkers (Table 2). HMWHA treatment was associated with faster discharge in patients with CXCL10 or PC1 values above median. Notably, these are the groups associated with worse outcomes. We then ran Cox proportional hazard models with interaction terms between biomarker (i.e., CXCL10, PC1, PC2) and treatment (HMWHA vs. placebo) for our outcomes and tested for Synergy Index (SI) between biomarker and treatment with and without controlling for covariates. We found a SI of 0.39, 0.31, and 0.51 for interactions with CXCL10, PC1 and PC2 scores, respectively, supporting that the effect of HMWHA on recovery may be modified by underlying inflammatory state (better outcomes in hyperinflammatory patients); these results, however, were not statistically significant and should be confirmed in larger cohorts.

## Discussion

Our results in aggregate support the utility of a combination of clinical and laboratory parameters to predict outcomes and response to treatment in severe viral pneumonia. We confirm that age and lung injury severity (leading to low PaO_2_/FiO_2_) are associated with poor outcomes in COVID19 and other pneumonias as previously reported [10]. In addition, our data support the use of CXCL10 as a predictor of outcomes and treatment responses in viral pneumonia. CXCL10 may be a marker for impaired T-cell immunity in viral pneumonia [11]. CXCL10 levels on hospital admission have been associated with severe pneumonia outcomes [12]. Interestingly, in our cohort CXCL10 performed as well, or better, as a panel including 6 additional inflammatory cytokines (IL6, CXCL8, CCL2, CCL3, IL10, and TNFα), suggesting that these other cytokines do not add predictive value to CXCL10 alone with regard to inflammatory response to COVID19. CXCL10 is however not specific for viral pneumonia, as it is upregulated by type I/II interferons, which can be activated by bacterial (e.g. tuberculosis), viral and non-infectious (e.g. autoimmune disease) processes [13]. Thus, it is best understood as a helpful biomarker of hyperinflammatory status, once the diagnosis of viral pneumonia is established.

Hyperinflammatory sub-phenotypes of ARDS and sepsis are associated with worse outcomes but are also more amenable to treatment interventions [14]. HMWHA can successfully treat acute severe COPD exacerbation and improves systemic markers of inflammation [6]. More recently, we showed that HMWHA improves viral pneumonia outcomes in humans and mice by ameliorating inflammation [7]. This analysis extends and refines previous findings by supporting that HMWHA treatment is associated with improved outcomes specifically in patients with elevated inflammatory markers. CXCL10 is produced by monocyte-derived macrophages in lung injury and is induced by HA fragments [15], which HMWHA efficiently inhibits [7].

In summary, this analysis underscores the need for inflammatory biomarker development as a predictive tool in severe viral pneumonia, enables targeted recruitment in future trials of HMWHA and other anti-inflammatory agents in viral pneumonia, and informs possible mechanistic hypotheses between treatment, biomarkers and outcomes, thus promoting precision therapy development.

**Table 1:** Multivariate Cox proportional hazard models for each biomarker and the recovery, time on supplemental oxygen, and time to discharge outcomes

	Recovery*			Time on supplemental oxygen			Time to discharge		
	HR	95% CI	p-value	HR	95% CI	p-value	HR	95% CI	p-value
*CXCL10 models*									
Age: > = 62 vs < 62 (reference)	0.56	(0.38, 0.83)	**0.004**	0.58	(0.38, 0.86)	**0.007**	0.57	(0.38, 0.85)	**0.005**
Gender: female vs male (reference)	1.07	(0.70, 1.62)	0.758	1.03	(0.67, 1.58)	0.881	1.06	(0.70, 1.62)	0.777
PaO_2_/FiO_2_: < 200 vs > = 200 (reference)	0.51	(0.34, 0.76)	**0.001**	0.53	(0.36, 0.79)	**0.002**	0.50	(0.33. 0.75)	**0.001**
CXCL10 median: Above vs below (reference)	0.68	(0.46, 1.01)	**0.053**	0.66	(0.45, 0.99)	**0.043**	0.73	(0.49, 1.08)	0.118
*PC1 models*									
Age: > = 62 vs < 62 (reference)	0.56	(0.38, 0.84)	**0.004**	0.58	(0.38, 0.86)	**0.008**	0.57	(0.38, 0.85)	**0.006**
Gender: female vs male (reference)	1.15	(0.76, 1.74)	0.513	1.13	(0.74, 1.72)	0.581	1.12	(0.74, 1.70)	0.603
PaO_2_/FiO_2_: < 200 vs > = 200 (reference)	0.52	(0.35, 0.77)	**0.001**	0.53	(0.36, 0.79)	**0.002**	0.50	(0.33, 0.75)	**0.001**
PC1 median: Above vs below (reference)	0.92	(0.63, 1.36)	0.681	0.91	(0.62, 1.35)	0.651	0.90	(0.60, 1.34)	0.602
*PC2 models*									
Age: > = 62 vs < 62 (reference)	0.58	(0.39, 0.86)	**0.006**	0.59	(0.39, 0.88)	**0.010**	0.57	(0.38, 0.86)	**0.007**
Gender: female vs male (reference)	1.11	(0.73, 1.67)	0.627	1.09	(0.72, 1.66)	0.672	1.10	(0.72, 1.68)	0.663
PaO_2_/FiO_2_: < 200 vs > = 200 (reference)	0.53	(0.36, 0.79)	**0.002**	0.55	(0.37, 0.81)	**0.003**	0.51	(0.34, 0.76)	**0.001**
PC2 median: Above vs below (reference)	1.39	(0.95, 2.03)	**0.088**	1.39	(0.95, 2.04)	**0.094**	1.15	(0.77, 1.71)	0.487

Nominally significant p-values are boldened. CI, confidence interval; HMWHA, high molecular weight hyaluronan; HR, hazard ratio; PC, principal component

*The recovery outcome is a composite of the time on supplemental oxygen and time to discharge outcomes

**Table 2:** Univariate Cox proportional hazard models for the recovery, time on supplemental oxygen, and time to discharge outcomes

Patient group		Recovery*			Time on supplemental oxygen			Time to discharge		
		HR	95% CI	p-value	HR	95% CI	p-value	HR	95% CI	p-value
*CXCL10*										
Placebo	CXCL10 upper vs lower median (reference)	0.619	(0.388, 0.988)	**0.045**	0.590	(0.364, 0.956)	**0.032**	0.678	(0.415, 1.107)	0.120
CXCL10 above median	HMWHA vs Placebo (reference)	1.479	(0.914, 2.395)	0.111	1.494	(0.913, 2.445)	0.110	1.581	(0.943, 2.653)	**0.083**
CXCL10 below median	HMWHA vs Placebo (reference)	0.937	(0.590, 1.489)	0.795	0.892	(0.558, 1.426)	0.633	1.065	(0.664, 1.707)	0.793
*PC1*										
Placebo	PC1 upper vs lower median (reference)	0.734	(0.463, 1.165)	0.189	0.700	(0.435, 1.126)	0.142	0.668	(0.411, 1.086)	0.104
PC1 above median	HMWHA vs Placebo (reference)	1.447	(0.914, 2.291)	0.115	1.397	(0.877, 2.226)	0.159	1.604	(0.991, 2.596)	**0.055**
PC1 below median	HMWHA vs Placebo (reference)	0.928	(0.576, 1.494)	0.758	0.906	(0.557, 1.475)	0.692	0.952	(0.581, 1.558)	0.844
*PC2*										
Placebo	PC2 upper vs lower median (reference)	1.540	(0.967, 2.454)	**0.069**	1.440	(0.891, 2.327)	0.137	1.325	(0.813, 2.160)	0.260
PC2 above median	HMWHA vs Placebo (reference)	0.980	(0.619, 1.550)	0.930	0.968	(0.610, 1.538)	0.892	1.112	(0.693, 1.785)	0.660
PC2 below median	HMWHA vs Placebo (reference)	1.344	(0.833, 2.168)	0.226	1.292	(0.789, 2.115)	0.309	1.426	(0.867, 2.346)	0.162

Nominally significant p-values are boldened. CI, confidence interval; HMWHA, high molecular weight hyaluronan; HR, hazard ratio; PC, principal component

*The recovery outcome is a composite of the time on supplemental oxygen and time to discharge outcomes

## Data Availability

Data are available from the corresponding author upon written request.

## References

[1] Virk S et al (2023) Comparing clinical outcomes of COVID-19 and influenza-induced acute respiratory distress syndrome: a propensity-matched analysis. Viruses 15(4):922 37112902 10.3390/v15040922 PMC10144713

[2] Cyphert JM, Trempus CS, Garantziotis S (2015) Size matters: molecular weight specificity of hyaluronan effects in cell biology. Int J Cell Biology 2015:563818 26448754 10.1155/2015/563818 PMC4581549

[3] Garantziotis S, Savani RC (2019) Hyaluronan biology: a complex balancing act of structure, function, location and context. Matrix Biol 78–79:1–10 30802498 10.1016/j.matbio.2019.02.002 PMC6774756

[4] Garantziotis S (2021) Modulation of hyaluronan signaling as a therapeutic target in human disease. Pharmacol Ther 232:107993 34587477 10.1016/j.pharmthera.2021.107993 PMC8930430

[5] Garantziotis S et al (2016) The role of hyaluronan in the pathobiology and treatment of respiratory disease. Am J Physiology-Lung Cell Mol Physiol 310(9):L785–L795 26747781 10.1152/ajplung.00168.2015 PMC4867348

[6] Galdi F et al (2021) Inhaled high molecular weight hyaluronan ameliorates respiratory failure in acute COPD exacerbation: a pilot study. Respir Res 22(1):30 33517896 10.1186/s12931-020-01610-x PMC7847749

[7] Stober VP et al (2025) Hyaluronan ameliorates viral pneumonia in mice and humans by inhibiting E2F1 transcription factor. Am J Respir Cell Mol Biol 10.1165/rcmb.2025-0173OC PMC12825446 40720792

[8] Pum A et al (2021) Cytokines and chemokines in SARS-CoV-2 infections-therapeutic strategies targeting cytokine storm. Biomolecules 11(1):91 33445810 10.3390/biom11010091 PMC7828218

[9] Beigel JH et al (2020) Remdesivir for the treatment of Covid-19-final report. N Engl J Med 383(19):1813–1826 32445440 10.1056/NEJMoa2007764 PMC7262788

[10] Bassetti M et al (2020) Balancing evidence and frontline experience in the early phases of the COVID-19 pandemic: current position of the Italian society of Anti-infective therapy (SITA) and the Italian society of pulmonology (SIP). Clin Microbiol Infect 26(7):880–894 32360444 10.1016/j.cmi.2020.04.031 PMC7195088

[11] Rydyznski Moderbacher C et al (2020) Antigen-Specific adaptive immunity to SARS-CoV-2 in acute COVID-19 and associations with age and disease severity. Cell 183(4):996–1012e19 33010815 10.1016/j.cell.2020.09.038 PMC7494270

[12] Lore NI et al (2021) CXCL10 levels at hospital admission predict COVID-19 outcome: hierarchical assessment of 53 putative inflammatory biomarkers in an observational study. Mol Med 27(1):129 34663207 10.1186/s10020-021-00390-4 PMC8521494

[13] Berry CM, Hertzog PJ, Mangan NE (2012) Interferons as biomarkers and effectors: lessons learned from animal models. Biomark Med 6(2):159–176 22448790 10.2217/bmm.12.10

[14] Sinha P et al (2023) Identifying molecular phenotypes in sepsis: an analysis of two prospective observational cohorts and secondary analysis of two randomised controlled trials. Lancet Respir Med 11(11):965–974 37633303 10.1016/S2213-2600(23)00237-0 PMC10841178

[15] Tighe RM et al (2011) Recruited exudative macrophages selectively produce CXCL10 after noninfectious lung injury. Am J Respir Cell Mol Biol 45(4):781–788 21330464 10.1165/rcmb.2010-0471OC PMC3208617

